# Imogolite Reinforced Nanocomposites: Multifaceted Green Materials

**DOI:** 10.3390/ma3031709

**Published:** 2010-03-09

**Authors:** Weng On Yah, Kazuya Yamamoto, Nattha Jiravanichanun, Hideyuki Otsuka, Atsushi Takahara

**Affiliations:** 1 Graduate School of Engineering, Kyushu University, 744 Mootoka, Nishi-ku, Fukuoka 819-0395, Japan; E-Mail: wo-yah@cstf.kyushu-u.ac.jp (W.O.Y); 2 Kitakyushu College of Technology, 5-20-1 Shii, Kokuraminami, Kitakyusyu, Fukuoka 802-0985, Japan; E-Mail: kyamamot@kct.ac.jp (K.Y); 3 Institute for Materials Chemistry and Engineering, Kyushu University, 744 Mootoka, Nishi-ku, Fukuoka 819-0395, Japan; E-Mails: may@cstf.kyushu-u.ac.jp (N.J); otsuka@ms.ifoc.kyushu-u.ac.jp (H.O)

**Keywords:** imogolite, nanotube, nanocomposite, surface modification, *in situ* synthesis, spin-assembly

## Abstract

This paper presents an overview on recent developments of imogolite reinforced nanocomposites, including fundamental structure, synthesis/purification of imogolite, physicochemical properties of nanocomposites and potential applications in industry. The naturally derived nanotubular material of imogolite represents a distinctive class of nanofiller for industrially significant polymer. The incompatibility between the surface properties of inorganic nanofiller and organic matrix has prompted the need to surface modify the imogolite. Early problems in increasing the binding properties of surface modifier to imogolite have been overcome by using a phosphonic acid group. Different approaches have been used to gain better control over the dispersal of nanofiller and to further improve the physicochemical properties of nanocomposites. Among these, polymer grafting, *in situ* synthesis of imogolite in polymer matrix, and spin-assembly are some of the promising methods that will be described herein. This imogolite reinforced nanocomposite of enhanced optical and mechanical properties, and with unique biological and electronic properties, is expected to become an important category of hybrid material that shows potential for industrial applications.

## 1. Introduction

Nanotubular materials are the fundamental building blocks of future nanodevices for novel applications [1]. Recent scientific research in this area has brought significant progress in the field of catalysis, separation, sensing, biotechnology, electronics, energy conversion and storage. One such kind of well-studied nanotubular material from the fullerene family is known as carbon nanotube, which was discovered by Iijima *et al.* in 1991 [2]. Carbon nanotube, based on its excellent mechanical properties and electrical and thermal conductivity, has stimulated extensive research with a considerable number of studies focusing on developing high performance polymeric nanocomposites. Carbon nanotube is regarded as an ideal reinforcement nanofiller for polymer owing to its ability to enhance physicochemical properties, and has been increasingly investigated for potential nanotechnological applications. However, conventional techniques to produce sizeable amounts of carbon nanotube require energy intensive processes and extreme synthesis conditions, which from the industrial point of view is not feasible for widespread application. Therefore, to fully exploit the advantage of nanotubular material, researchers are eager to find a competent substitution for carbon nanotube. A naturally occurring single wall nanotube material consisting of hydrous aluminosilicate was found in 1962 in the weathered pumice bed of volcanic ash soil in the southern part of Kyushu, Japan [3]. This hydrous aluminosilicate material with the general formula [Al_2_O_3_·SiO_2_·2H_2_O] was called imogolite, named after the imogo soil of Kumamoto prefecture in which it occurs. Imogolite, like carbon nanotube, has high aspect ratio and surface area with length runs from several hundred nanometers to micrometers [4]. The physicochemical properties unique to imogolite are expected to have potential nanotechnological applications in catalysts, membrane, and adsorbents [5,6,7]. However, there are some problems in imogolite nanotechnology that remains to be overcome, for instance, the uniform dispersion of imogolite inside polymer matrix, tunability of imogolite dimensions (diameter and length), and interfacial adhesion between nanotube and polymer matrix.

The dispersion of imogolite is probably the most pressing issue to be resolved for the composite application [8]. The uniform dispersion of imogolite inside polymer matrix is very important from the perspective of nanomaterial engineering since it governs the macroscopic performance of the resulting polymeric nanocomposites. The imogolite nanotube should be individually distributed inside the polymer matrix to ensure an efficient load transfer to imogolite network. Moreover, a well dispersed imogolite will also impart transparency to nanocomposites, since an evenly dispersed imogolite nanotube will allow better transmission of light [9]. Hence, concentration of imogolite inside the polymer matrix should be controlled. High concentration of imogolite will force the nanotube to hold together into bundles *via* hydrogen bonding and van der Waal forces, which cause it to aggregate [10]. The dimension of the nanotube (length, L) is among the two key parameters that decide fracture mechanism as well as ultimate toughness and strength for this type of nanotube-reinforced nanocomposites; the other parameter being the interfacial shear strength (τ) between the matrix and the filler [11]. The macroscopic strength of nanocomposites is inversely proportional to nanotube length according to classical models of conventional uniaxial fiber reinforced composites [12]. In a case where L > L_c_ (critical length, ranges from circa 4 to 40 μm), a nanotube will easily be broken apart upon deformation of nanocomposites when transferred stress exceeds the nanotube fracture strength leading to nanocomposites failure. With this in mind, the length of imogolite should be tuned below the critical length to reduce nanotube fracture for higher transferred stress endurance [13]. There are numerous studies that report that surface treatment or chemical functionalization of inorganic nanotube is an indispensable step toward better interfacial adhesion between hydrophilic inorganic nanofiller and hydrophobic polymer matrices [14,15,16]. The surface of inorganic nanofiller usually consists of hydrophilic groups, which should be modified with organic moieties to increase the miscibility with the polymer matrix. For improved mixing, surface modified inorganic nanofillers are subjected to *in situ* polymerization, where the monomer is introduced into the nanofiller interval space so that the polymer formation can occur inside [17].

Apart from imogolite, some inorganic nanotubular material of halloysite [18], hydroxyapatite [19], boehmite [20], boron nitride [21], metal disulfides (NbS_2_, MoS_2_, WS_2_, TiS_2_, ZrS_2_, TaS_2_, ReS_2_, and HfS_2_) [22,23,24,25], and metal oxide (MgO, TiO_2_, SiO_2_, VO_x_, CeO_2_, Co_3_O_4_, and ZnCr_2_O_4_) [26,27,28,29,30,31] have been reported. Due to the unique properties of these inorganic nanotubular materials, considerable studies have been done to investigate the reinforcing effect for polymer matrix through hybridization and incorporation. Liu *et al.* [32] found that incorporation of silane-modified halloysite into epoxy resin significantly improved the flexural strength, char yield and dimensional stability of the resulting nanocomposites. The enhanced properties of this nanocomposite correlated with the intimate mixing of uniformly dispersed halloysite with epoxy resin. In fact, halloysite is a multilayer tubular aluminosilicate material analogous to imogolite, with a relatively larger internal diameter of 15 nm and length of 0.5–1 μm. Interestingly, halloysite nanotubes have surface chemistry opposite to that of imogolite, *i.e.*, aluminum hydroxide on internal and silicate on external surfaces. Halloysite has been studied extensively by Lvov′s group, who successfully used it into multifunctional nanometer-scale containers that can be applied as effective drug release agents, biomimetic reaction vessels, additives for protective coatings, *etc.* [33,34,35,36]. On the other hand, works done by Lu *et al.* [37] and Cheng *et al.* [38] indicate that nanotubular titania is an attractive option as an electron acceptor over irregular nanoparticles in terms of electron transport for nanocomposites photovoltaic cells after combination with poly(3-hexylthiophene) and polyaniline. Electron transport in titania nanotubes was reported to be much more efficient than that of titania nanoparticles owing to the one-dimensional nanostructure and larger interfacial area. Excellent properties afforded by the synergistic effect of combining silica tubes with polyimide were reported by Fu et al [39]. The overall performance of composites, e.g., tensile strength, ductility, tearing strength, volume and surface resistivity have been significantly improved by introduction of low silica tube content. Interestingly, the silica tubes/polyimide composite exhibits lower density compared to the pristine polyimide. Up until now, nanotube-reinforced nanocomposites were mostly based on carbon nanotubes. However, carbon nanotube, and some inorganic nanotubes reported, are artificial materials involving an expensive and time-consuming preparation processes. Generally, these materials are opaque (due to conjugated π-system of carbon nanotube and energy band gap of some transition metals), which might not be suitable for certain optical applications. In addition, issues concerning their potential toxicity to humans and the environment have become a stumbling block to the development and application of these nanotube-reinforced nanocomposites.

In this respect, imogolite, a low cost and naturally available green nanotube, with its high aspect ratio, surface area and inherited transparent properties [40], is worthy to be explored as a versatile nanofiller for an industrially significant polymer. In this paper, a review of recent progress on imogolite-based nanocomposites and hybrid materials carried out by our group is presented. These imogolite-reinforced nanocomposites are expected to become an important category of hybrid material that shows promising industrial applications.

## 2. Imogolite

**Figure 1:**
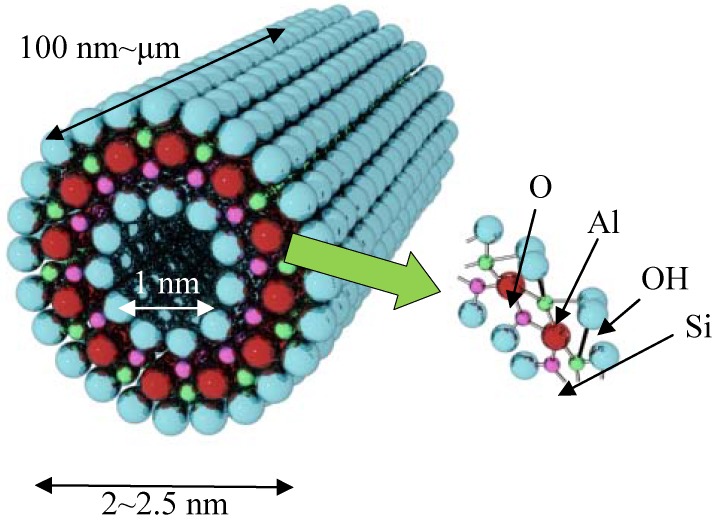
Schematic representation of imogolite nanotube structure.

**Figure 2:**
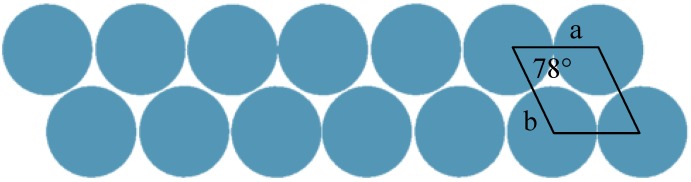
Monoclinic solid-state packing arrangement of the imogolite nanotubes.

Discovery of imogolite can be traced back as early as 1962. Investigation of the material by Cradwick *et al.* in 1972 confirmed its composition [Al_2_O_3_·SiO_2_·2H_2_O] and atomic coordination provided by electron diffraction analysis [4]. Complete study of imogolite porosity based on N_2_, CO_2_, and CH_4_ adsorption data by Ackerman *et al.* [41] found that imogolite possesses an inner pore diameter of 1 nm. The schematic representation of imogolite is shown in Figure 1. Cradwick suggested that the wall structure of imogolite consists of a layer of aluminum (III) hydroxide (gibbsite) that lies on the outer wall, with silicate groups dangled on the inner wall [4]. It was observed that the imogolite tubes pack on each other in a hexagonal close packing arrangement *via* hydrogen bonds that form between the tubes [42]. The latest crystallographic study, however, has shown that well ordered solid state packing of imogolite tubes can only be achieved under monoclinic arrangement (Figure 2) [43]. In 1977, Farmer *et al.* proposed a preparation method to artificially synthesize imogolite from mild chemistry of Al(ClO_4_)_3_ and Si(OH)_4_ [44]. Though the synthesis method was developed three decades ago, the formation mechanism of imogolite is not well understood. Early study [45] suggested that the tubular morphology evolution is started by the binding of isolated silicate groups to the gibbsite sheet where the tetravalent silicon atoms pull the oxygen atoms of the gibbsite sheet into curvature cylinder (bond length of Al-O and Si-O is 0.19 nm and 0.16 nm, respectively). Early attempts to tune the imogolite dimensions (diameter) by changing the synthesis time were not successful, as the formation of nanotube occurred at an early stage and the structure does not change significantly throughout the synthesis process [43]. Nevertheless, recent works by Su *et al.* have shed light on clarifying the imogolite growth mechanism. Using a newly developed Transmission Electron Microscopy (TEM) specimen preparation method *via* droplet evaporation technique to individually disperse imogolite nanotubes [46], semiquantitative analysis of tube number and dimensions was made possible. Detailed analysis of the numbers, lengths, and length distribution of imogolite nanotubes support the kinetic growth mechanism [47]. The TEM results were consistent with this mechanism where protoimogolites and short imogolite are observed in the initial stage of synthesis and average length of the nanotubes increased rapidly with reaction time. Moreover, since the end of the imogolite nanotube stays open and active, the synthesized nanotube can continue to grow in replenished precursor solution.

## 3. Imogolite as a Natural and Artificial Nanotubular Material

**Figure 3:**
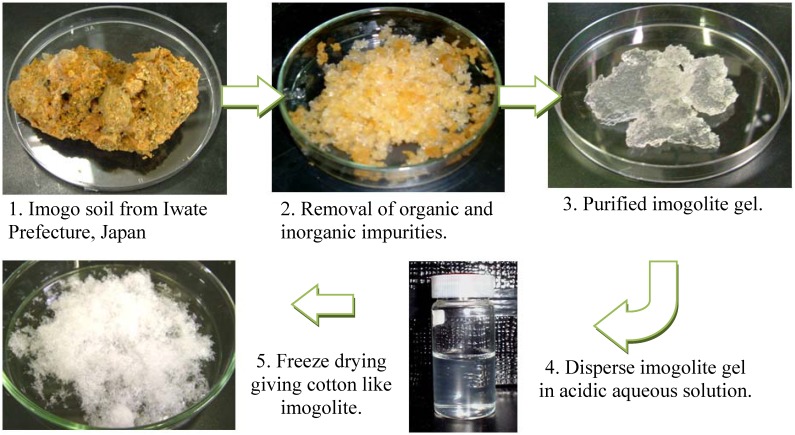
Purification steps of imogolite from imogo soil.

Naturally occurred imogolite derived from glassy volcanic ash soil (imogo soil) normally appears as brown-red in color due to impurities of metal oxides and hydroxides. These impurities can be removed by purification as described [48]. Basically, filmy gel containing the imogolite (collected from Kitakami, Iwate, Japan) is suspended in water and ultra-sonicated repeatedly. Occluded organic and inorganic impurities (iron and manganese oxide) are removed by treating the gel with hot 1.8 M H_2_O_2_, followed by citrate-bicarbonate (DCB). The gel is further treated with cold 0.5 M Na_2_CO_3_ to wash away citrate remnants. Purified imogolite gel is redispersed in weak acidic solution and ultra-sonicated for a few hours. Freeze drying of the solution will result in cotton-like imogolite. Figure 3 illustrates the purification step to recover the imogolite from imogo soil.

Farmer *et al.* made the first report on artificial synthesis of imogolite in 1977 [44]. The general preparation method is as follows: Tetraethoxysilane aqueous solution is added to an aqueous solution of aluminum chloride (AlCl_3_·6H_2_O). The resulting solution contains 2.4 mM of Al and 1.4 mM of Si. The pH is adjusted to 5.0 by dropwise addition of 0.1 M of NaOH solution followed by 1 mmol of HCl and 2 mmol of acetic acid per liter of the solution. The pH adjusted solution is refluxed under 369 K for 120 h and cooled to room temperature. The suspended material is gelated with NaCl solution, washed with double-distilled water, collected by filtration, and redispersed again in low acidic solution. The final step involves freeze drying the solution to retrieve the cotton like imogolite. The final freeze dried imogolite product will undergo surface modification prior to incorporation into polymer matrix.

## 4. (Imogolite/PMMA) Nanocomposites *via* Polymer Grafting through Method

As mentioned previously, the dispersion of inorganic nanofiller inside the polymer matrix is crucial for high performance nanocomposites. The degree of dispersion of nanofiller depends strongly on the surface properties and interfacial compatibility between the inorganic and organic components. Several works have been done to convert the surface property of nanofiller by grafting with coupling agents such as organosilanes [49]. Pinnavaia *et al.* proposed (γ-aminopropyl)triethoxysilane (APS) as a surface modifier for imogolite. Both FTIR and ^29^Si MAS NMR data indicate that APS was successfully grafted onto imogolite Al-OH surface. However, this APS-imogolite bond is labile under hydrated conditions, which complicates the use of this silylated nanotubular material as nanofiller for polymeric nanocomposites [50]. Despite the daunting fact that hydrolysis occurred on APS-imogolite interface, APS was still used as coupling agent for other inorganic nanotubular material [51].

**Figure 4:**
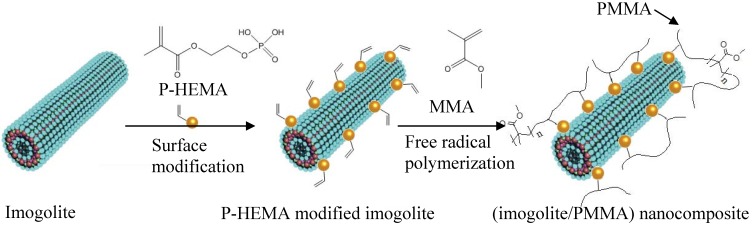
Chemical structure of 2-acidphosphoxyethyl methylacrylate (P-HEMA) and preparation of (imogolite/PMMA) nanocomposite using P-HEMA-modified imogolite as a nanofiller.

A more convenient and facile approach is being studied that employs organophosphonic acid groups as a surface modifier for aluminosilicate imogolite. This organophosphonic acid group has been shown to exhibit favorable binding properties over Al-OH, and thus, widely used as a protective layer for Al-sheet [52]. Our group utilized octadecylphosphonic acid (OPA) as a surface modifier for imogolite [53]. The OPA-modified imogolite showed excellent dispersion in an aprotic organic solvent of chloroform and hexane. Essentially, good dispersion of imogolite in organic solvent is crucial to facilitate uniform dispersion of nanofiller in hydrophobic polymer matrix. Nevertheless, having a hydrophobic imogolite is not enough to achieve tough polymeric nanocomposites, since the nanofiller are only holding the polymer through weak van der Waal interactions. As a result, detachment of nanofiller from polymer matrix takes place due to unsatisfactory interfacial interaction. One strategy used to increase the imogolite dispersal and strengthen the interaction with the polymer matrix is to place a polymerizable functional group on organophosphonic acid. This distinctive organophosphonic acid not only acts as a surface modifier for imogolite, but also serves as an additional monomer source for polymerization. In the case of preparing high performance (imogolite/PMMA) nanocomposite, 2-acidphosphoxyethyl methacrylate (P-HEMA) with a polymerizable methacrylate group was proposed as a surface modifier for imogolite [54]. P-HEMA-modified imogolite was mixed together with methyl methacrylate monomer (MMA) so that subsequent free radical polymerization could occur on both P-HEMA and MMA, eventually giving a better interaction between imogolite and the resulting PMMA. Figure 4 schematically illustrates the preparation process of (imogolite/PMMA) nanocomposites together with the structure of P-HEMA by grafting through method. The formation of the P-HEMA-imogolite interaction can be revealed by the FT-IR method.

**Figure 5:**
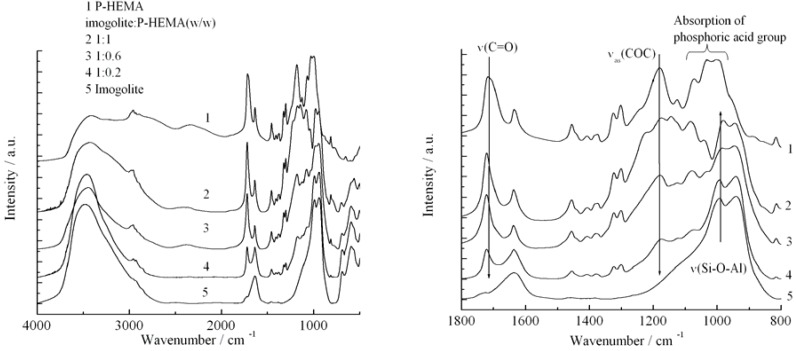
FT-IR spectra of P-HEMA, imogolite, and imogolite/P-HEMA at various P-HEMA contents in the full scale (left) and the region from 1800 to 800 cm^-1^ (right). Reprinted with permission from Ref. [54]. Copyright 2005 Elsevier B. V.

Figure 5 shows the FT-IR spectra of (1) P-HEMA, (2)-(4) P-HEMA-modified imogolite with different P-HEMA concentrations (imogolite/P-HEMA), and (5) natural imogolite. Both spectra (1) and (5) show the characteristic IR absorption of pristine P-HEMA and imogolite, respectively. The absorption bands of the distinct functional groups of P-HEMA and imogolite appeared in the IR spectra of imogolite/P-HEMA hybrids indicating the successful surface modification process on imogolite by P-HEMA. The intensity of υ_as_(PO_3_^2-^) absorption band at 1080 cm^-1^ increased with the increasing concentration of P-HEMA in the hybrid, confirming the absorption of P-HEMA on the imogolite surface. The position of υ_s_(PO_3_^2-^) bands at 995 cm^-1^ shifted to 982 cm^-1^ and 987 cm^-1^ for 1:1 and 1:0.6, respectively (the band shift was undetectable for 1:0.2). This shifting is due to the alteration of υ_s_(PO_3_^2-^) bonding when interacting with the O-H group on the imogolite surface. The FT-IR data were consistent with previous experiments, which reported that an absorbed P-HEMA on substrate generally has υ_s_(PO_3_^2-^) absorption of around 980 cm^-1^, thus providing strong evidence that the P-HEMA was assembled on the imogolite surface [55]. The amounts of the adsorbed P-HEMA on the imogolite surface were estimated by elemental analysis and found to be 0.15, 0.36, and 0.51 mg per 1 mg of imogolite (corresponds to imogolite:P-HEMA =1:0.2, 1:0.6, and 1:1). The surface coverage of P-HEMA on imogolite was calculated to be 13.8, 33.5, and 47.5% by assuming complete modification of P-HEMA on the imogolite nanotube.

**Figure 6:**
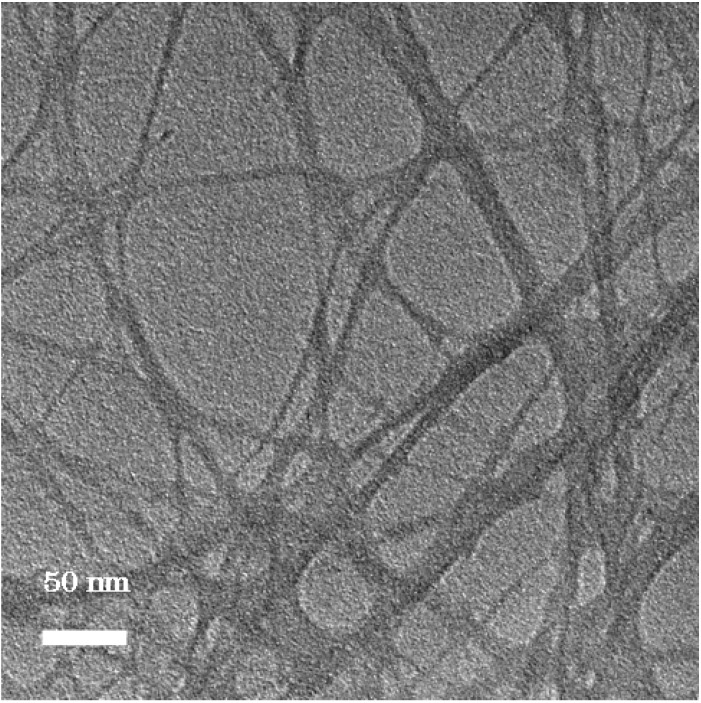
TEM image of P-HEMA-modified imogolite. Reprinted with permission from Ref. [54]. Copyright 2005 Elsevier B. V.

**Figure 7:**
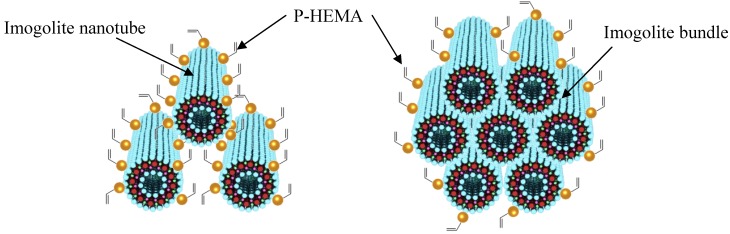
Adsorption of P-HEMA on a single imogolite nanotube (left) and adsorption to a bundle of imogolite nanotubes (right).

The image of P-HEMA-modified imogolite visualized by TEM is shown in Figure 6. The morphology of P-HEMA-modified imogolite was identical to the unmodified one with diameters ranging from circa 5−25 nm. The diameter of the observed P-HEMA-modified imogolite was larger than the diameter of single imogolite nanotube of circa 2 nm. It was suspected that the P-HEMA was intercalated inside imogolite bundle, thus expanding the imogolite nanotube interval distance (Figure 7, left). This can be explained by the wrapping of P-HEMA on imogolite nanotube, with larger P-HEMA-modified imogolite diameter corresponding to a higher extent of P-HEMA absorption. However, such diameter enlargement is hardly being judged to ascribe to intercalation or wrapping of P-HEMA because of the poor contrast between the absorbed P-HEMA and imogolite nanotube. Rather, the plausible reason for this phenomenon would be the aggregation of imogolite nanotube into bundles driven by strong hydrogen bonding interactions, followed by low levels of P-HEMA adsorption on the imogolite bundle surface (Figure 7, right). Despite that the phosphonic acid group is said to have relatively stronger binding properties to Al-OH, it is hard to rule out that the aggregation of surface-modified imogolite is impossible to happen. It is important to note that imogolite nanotubes appears in the bundle formation at an early stage, and any attempt to split them apart into single nanotubes is unattainable. However, one should focus on controlling the extent of imogolite aggregation, so that the diameter of the imogolite bundle is kept within nano-scale level. Judging from the TEM image, it can be approximated that a single imogolite bundle of 5−25 nm diameter accommodated 2 to 12 imogolite nanotubes. P-HEMA had thus achieved the purpose of controlling the aggregation of imogolite nanotubes without forming larger particulate by keeping the diameter of the imogolite bundle well below 50 nm. This observation is also supported by the dispersion test of P-HEMA-modified imogolite in hydrophobic solvent, where good dispersal of modified imogolite lasts days without flocculation or precipitation.

For the preparation of PMMA/imogolite nanocomposite, modified imogolite with the highest amount of absorbed P-HEMA (imogolite:P-HEMA = 1:1) was used for subsequent *in situ* MMA polymerization reaction. Since imogolite/P-HEMA of 1:1 has the highest P-HEMA coverage on imogolite surface, it is expected to have higher hydrophobicity, which facilitates better dispersion in MMA solution. The *in situ* polymerization process of MMA with P-HEMA-modified imogolite was confirmed by both NMR and FT-IR, with a conversion rate of 95−98% supported by NMR data. Also, GPC data shown in Table 1 indicates that radical polymerization of MMA proceeded smoothly with the presence of 1 wt % of either modified or unmodified imogolite.

**Table 1:** Molecular weight and polydispersity index of soluble polymer contents in PMMA/imogolite nanocomposite. ^a^

Run No.	Imogolite content (wt %)	M_n_	M_w_/M_n_	Type of imogolite
1	0	58,800	2.50	-
2	1.0	65,100	2.55	Modified
3	1.0	65,600	2.54	Unmodified

^a^ Only soluble part through 0.5 μm size filter was measured.

**Figure 8:**
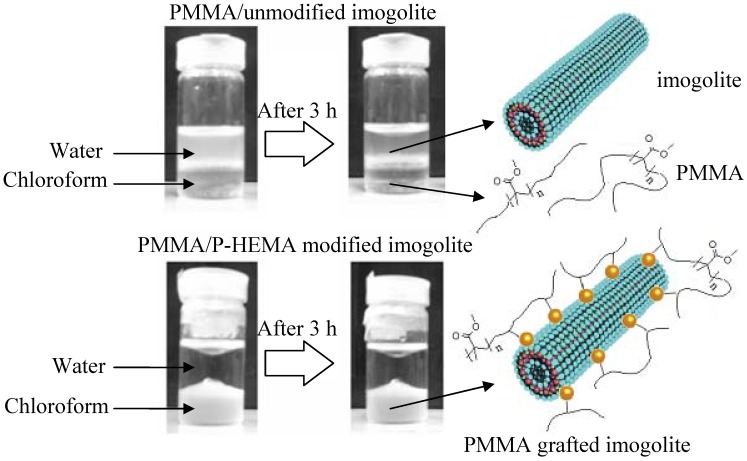
Dispersion state of PMMA grafted imogolite and unmodified imogolite in water/chloroform biphasic mixture. Reprinted with the permission from Ref. [54]. Copyright 2005 Elsevier B. V.

Both samples of PMMA polymerized with the presence of P-HEMA modified and unmodified imogolite were subjected to the dispersion test in a solution of water and chloroform biphasic mixture (Figure 8). For the PMMA/unmodified imogolite sample, it is obvious that the solvent phase remained clear (chloroform is a good solvent for PMMA) while the aqueous phase become slightly turbid, probably due to the suspension of detached imogolite. On the other hand, the aqueous phase of the PMMA/P-HEMA modified imogolite sample remained clear, with no sign of imogolite suspension observed. However, dispersion of the PMMA/P-HEMA imogolite in the chloroform phase caused the solvent to become highly turbid and the turbidity remained for days without precipitation. The contrast appearance of clear aqueous solution and turbid chloroform solvent implied that the P-HEMA modified imogolite, which fully incorporated into the PMMA polymer network, dissolved well in the organic phase with no detachment of nanofiller occurring like as the one in the sample of PMMA/unmodified imogolite. Next, a comparative study on optical and mechanical properties was carried out between the PMMA polymerized with P-HEMA modified imogolite (PMMA/imogolite nanocomposite) and PMMA mixed with unmodified imogolite *via* simple solution blend (PMMA/imogolite blend).

**Figure 9:**
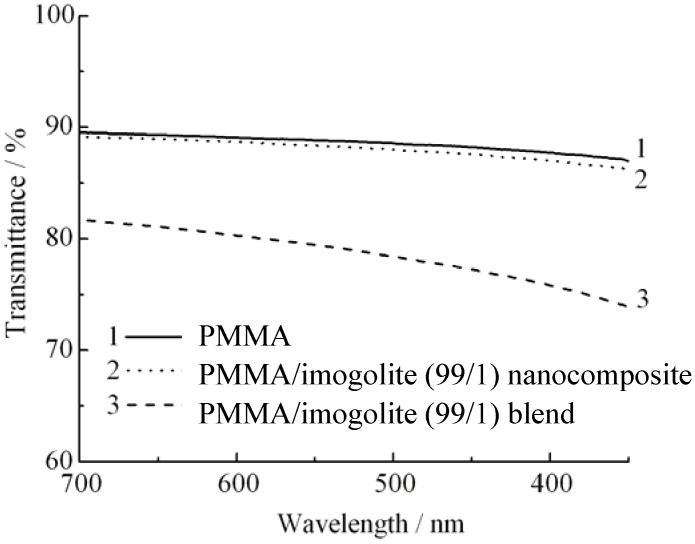
Light transmittance of PMMA, PMMA/imogolite nanocomposite, and PMMA/imogolite blend films. Reprinted with permission from Ref. [54]. Copyright 2005 Elsevier B. V.

PMMA, a transparent industrial polymer capable of transmitting visible light up to 92%, is widely used as a substitution to glass. Depending upon the dispersion state of nanofiller, incorporation of nanofiller usually affects the transparency of the PMMA, resulting in an optical loss to the PMMA nanocomposites. This is because non-uniform dispersion of nanofiller tends to aggregate into macroscopic size filler, which scatters light and decreases the optical quality of nanocomposite produced. Hence, without waiving PMMA transparency properties, uniform dispersion of nanofiller should be stressed when preparing the nanocomposites. As for the PMMA based nanocomposites, a noticeable clarity difference is observed for samples prepared by two different methods of *in situ* polymerization and solution blend. As can be examined from the light transmittance spectra (Figure 9) of pristine PMMA, 1 wt % of imogolite content of (PMMA/imogolite nanocomposite), and (PMMA/imogolite blend); simple blending of PMMA with imogolite caused almost 10% loss in light transmittance. However the transparency of (PMMA/imogolite nanocomposite) is almost identical to the pristine PMMA with no significant lost of light transmittance being detected. This result shows that PMMA/imogolite nanocomposite prepared *via* *in situ* polymerization has much more uniform nanofiller dispersal compared to the solution blend sample. Despite the inherent transparency of imogolite, solution blending of imogolite in PMMA *via* simple mechanical agitation method induces aggregation of imogolite, turning it into particles large enough to scatter visible light in the wavelength region of 300 nm to 700 nm.

Quantitative results, which provide a very descriptive detail of the optical properties of PMMA, PMMA/imogolite nanocomposite and PMMA/imogolite blend, based on transmittance and haze value is tabulated in Table 2. Generally, the lower the haze value, the less light the material can scatter and the less haziness it has. This haziness, which is an indicator of the formation of imogolite particulate, is measured by D65 light source with the value averaged at five different spots on the film. As can be seen in Table 2, the haze value of PMMA/imogolite blend was higher than that of the PMMA/imogolite nanocomposites. This raises the speculation that larger particulate imogolite is formed from the blend sample. These observations are in good agreement with the earlier findings and provide some insight on how the dispersal of imogolite nanotubes can have a dramatic effect on the optical properties of nanocomposites.

**Table 2:** Haze value and transmittance of PMMA, PMMA/imogolite nanocomposite, and PMMA/imogolite blend. ^a^

Sample	Haze value	Transmittance/% ^b^
PMMA	0.59 ± 0.11	88.6
PMMA/imogolite (99/1) nanocomposite	1.18 ± 0.07	88.0
PMMA/imogolite (99/1) blend	3.37 ± 0.56	78.6

^a^ Imogolite content in polymer matrix was 1.0 wt %. ^b^ Detected by light transmittance measurement at λ = 500 nm.

**Figure 10:**
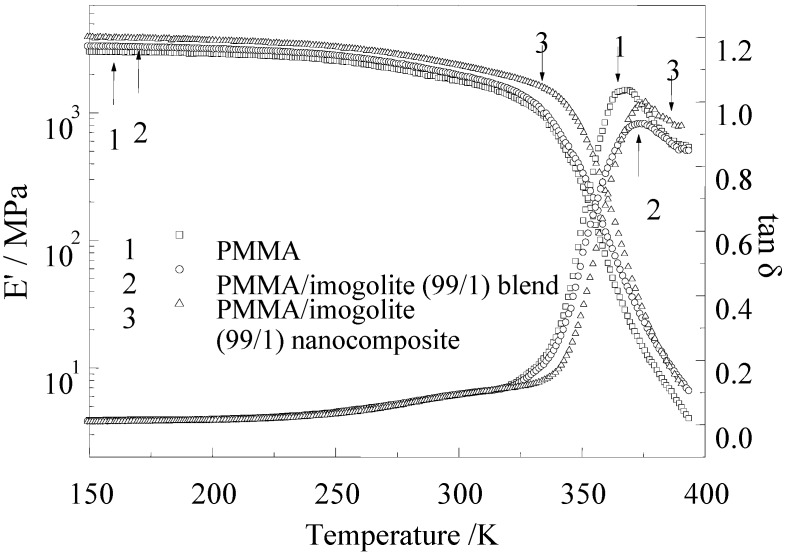
Temperature dependence of dynamic storage modulus and loss tangent at 11 Hz for PMMA, PMMA/imogolite blend, and PMMA/imogolite hybrid films with 1.0 wt % imogolite. Reprinted with permission from Ref. [54]. Copyright 2005 Elsevier B. V.

The dynamic mechanical properties of PMMA, with its nanocomposite and blend sample, were evaluated by dynamic viscoelasticity measurement. Figure 10 shows the temperature dependence of storage modulus (E^'^) and loss tangent (tan δ) for pristine PMMA, PMMA/imogolite blend, and PMMA/imogolite nanocomposite. As can be observed in Figure 10, the storage modulus of PMMA/imogolite blend decreased rapidly with increasing temperature starting from 300 °C, in a similar manner to pristine PMMA. On the other hand, PMMA/imogolite nanocomposite film exhibited E' values of 1.5-times that of pristine PMMA film over the entire temperature range investigated. The result shows that little or no significant interaction existed between the PMMA and unmodified imogolite interface for PMMA/imogolite blend sample. In contrast, covalent bond formed between the PMMA and P-HEMA of imogolite, restricting molecular mobility in the interface region, which resulted in shifting this storage modulus and loss tangent of PMMA/imogolite nanocomposite to a higher temperature region. The measured tensile modulus, strength and elongation of PMMA, PMMA/imogolite nanocomposite and PMMA/imogolite blend obtained from tensile test are listed in Table 3. It is obvious that incorporation of imogolite shows improved reinforcement effect for PMMA, since the nanocomposite and blend sample give a higher tensile modulus and ultimate strength than that of pristine PMMA. The strengthening effect is intensified when P-HEMA-modified imogolite was utilized as nanofiller for PMMA/imogolite nanocomposite, with both tensile modulus and ultimate strength 1.4-times higher than for the pristine PMMA film. Nevertheless, incorporation of imogolite dramatically diminished the stretch ability as evidenced by the lower elongation at break for both PMMA/imogolite nanocomposite and PMMA/imogolite blend. PMMA/imogolite blend, in particular, had the lowest elongation among the samples, probably due to irreversible slipping occurring between PMMA and the imogolite surface upon mechanical deformation. The mechanical properties complement the previous result, which demonstrates the importance of the dispersal of imogolite and the interfacial interaction in determining the performance of the nanotube-reinforced nanocomposite material.

**Table 3:** Tensile modulus, strength, and elongation of PMMA, PMMA/imogolite nanocomposite, and PMMA/imogolite blend films evaluated by tensile test. ^a^

Sample	Modulus/GPa	Strength/MPa	Elongation/%
PMMA	1.13 ± 0.05	27.6 ± 0.6	38.0 ± 13.7
PMMA/imogolite (99/1) nanocomposite	1.55 ± 0.05	38.5 ± 0.8	21.0 ± 8.9
PMMA/imogolite (99/1) blend	1.20 ± 0.08	27.7 ± 1.2	5.0 ± 1.2

^a^ The imogolite content in polymer matrix was 1.0 wt %.

## 5. (Imogolite/PVA) Nanocomposites *via* *in Situ* Imogolite Synthesis Method

Imogolite of one-dimensional aluminosilicate material, with its excellent thermal and mechanical properties, is expected to become a versatile reinforcement material for polymer. For this purpose, imogolite should be uniformly dispersed within the polymer matrix, which would satisfy both efficient load transfer to imogolite and higher optical transparency of nanocomposites, as demonstrated in the previous section. Another feasible approach to overcome the aggregation would be *in situ* synthesis of imogolite on the exact sites where they should be present within the polymer matrix [56]. As a way of manipulating the imogolite morphology, hydrothermal reactions involving aqueous solution shows some advantages of performing this key task. The success of *in situ* synthesis of nanofiller was verified by few nanocomposites systems [57,58], for instance, Lu *et al.* has successfully created homogeneous and reproducible fullerene C_60_ nanorods *in situ* within the P3HT matrix using controlled solvent vapor treatment (C-SVT) [58]. The P3HT/C_60_ nanorod composite showed better power conversion efficiency compared to the devices without optimization. The precise control over the homogeneity and specific size of C_60_ by *in situ* synthesis has become a novel methodology that can be extended to other types of nanofiller/polymer systems for higher performance capability.

Owing to the compatibility of imogolite with OH groups of organic moieties, water soluble polymer of poly(vinyl alcohol) (PVA) were used here as an example to show the feasibility of *in situ* synthesis of uniform imogolite nanotubes *via* hydrothermal reaction. PVA is a semicrystalline polymer with molecular a chain in the crystal region identical to that of polyethylene. Due to the conformation similarity with polyethylene, the crystal lattice modulus of PVA is estimated to be over 250 GPa [59]. However, the film has a poor stretch ability due to inter- and intramolecular hydrogen bonding within hydroxyl groups, which has diminished tensile moduli and strength of PVA film. A preliminary study on the physicochemical properties of PVA blended with imogolite *via* solution cast method was reported by Hoshino *et al.*, who intended to investigate the lyotropic mesophase formation in PVA/imogolite composite [60]. The enhanced elastic modulus of this solution cast PVA/imogolite is insignificant, with both flexible PVA and non-uniform dispersed imogolite contributing no cooperative effect in mechanical properties. On the other hand, Choi *et al.* attempted to improve the mechanical properties of syndiotacticity-rich and ultra-high molecular weight PVA (UHMW PVA) by blending with imogolite [61]. Coiling of UHMW PVA chains along the imogolite axis, which increased the molecular orientation of the crystalline phase of UHMW PVA in blend film, resulted in a maximum tensile modulus of 19.8 GPa and maximum tensile strength of 1.8 GPa. Although the UHMW PVA has higher crystal lattice modulus with both mechanical and physical properties superior to atactic PVA, its poor solubility [61] limits its potential in some industrial applications.

**Figure 11:**
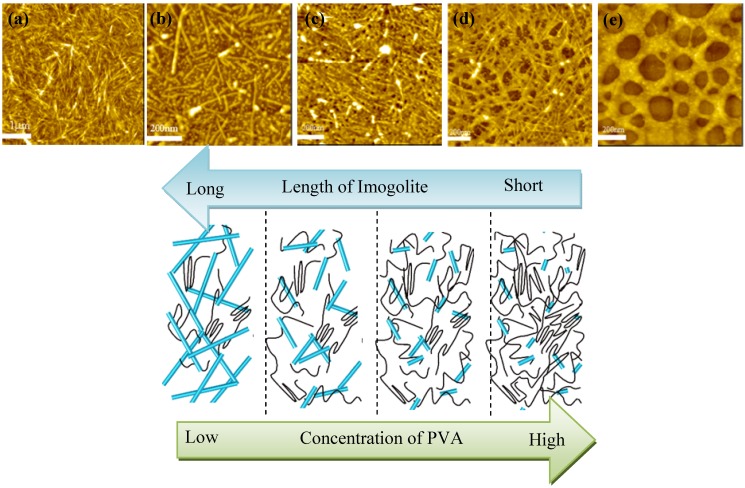
Cyclic contact mode AFM height images of *in situ* synthesized imogolite/PVA nanocomposites. Imogolite:PVA (w/w) = (a) 1:1, (b) 1:5, (c) 1:10, (d) 1:20, and (e) 1:50 (upper). Schematic illustrations of the size and length of imogolite nanotube achieved at the corresponding PVA concentration (bottom). Reprinted with permission from Ref. [56]. Copyright 2005 Royal Society of Chemistry.

Nonetheless, we would like to emphasize in this review that the focus of this work is to show the successful creation of imogolite nanotubes in the PVA matrix and the consequent optical/mechanical performance of the resulting nanocomposite. PVA with a degree of polymerization of 630 was mixed with pH-adjusted diluted Al/Si solution, and stirred at 369 K for 96 h. Mixed solutions with different mass ratio of (imogolite:PVA) 1:1, 1:5, 1:10, 1:20, and 1:50 were prepared. White product of imogolite/PVA nanocomposites were retrieved by precipitation of the solution by ethanol, filtration and rinsing thoroughly with water and ethanol. For optical and mechanical measurement, cast films with a thickness of 100 μm were also prepared [56]. FT-IR characterization of samples confirmed the successful *in situ* synthesis of imogolite that showed two sharp peaks at 995 and 930 cm^-1^ corresponding to Si-O-Al stretching vibration. Supplement WAXD data of nanocomposites showed characteristic reflection peaks of imogolite at 2.0, 0.98 and 0.33 nm [62] also proving that imogolite growed *in situ* in the PVA matrix. As revealed by the cyclic contact mode atomic force microscopy (AFM) images shown in Figure 11, except for imogolite/PVA with weight ratio of 1:50, all samples showed relatively well defined nanotubular morphology. (Note that the brighter area in the cyclic contact mode AFM images corresponds to the higher height region). This morphology indicates that randomly oriented imogolite dispersed homogeneously in the PVA matrix. The lengths of this *in situ* synthesized imogolite estimated by AFM images under various PVA concentrations were 689 ± 47 nm (imogolite:PVA = 1:1), 271 ± 21 nm (1:5), 224 ± 18 nm (1:10) and 178 ± 9 nm (1:20). As also schematically illustrated in Figure 11, the length of the obtained imogolite nanotube greatly depended on the concentration of PVA. A high concentration of PVA that forms strong interactions with the imogolite surface *via* hydrogen bonding will hinder the diffusion of Al/Si precursor to the imogolite surface. Gradually, the growing ends of imogolite have little free access to the precursor that provides nuclei to the growth and formation of nanotubes by polymerization, and eventually the imogolite will stop to grow when the Al/Si precursor is depleted. As in the case of imogolite:PVA = 1:50, the solution is too viscous for the precursor to acquire sufficient mobility to nucleate and grow into nanotube. Additionally, plausible bonding formed between the PVA and Al ion is also a leading factor inhibiting the growth and formation of imogolite nanotube, since a previous study has showed that PVA can react with metal salt in aqueous solution to form a metal chelate [63].

**Figure 12:**
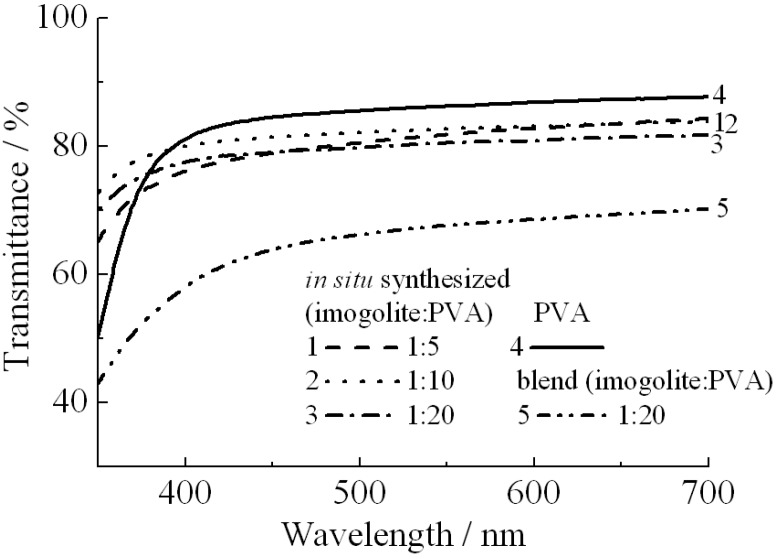
Light transmittance of *in situ* synthesized imogolite/PVA nanocomposite, imogolite/PVA blend, and PVA films. The magnitude of transmittance was corrected by film thickness. Reprinted with permission from Ref. [56]. Copyright 2005 Royal Society of Chemistry.

**Figure 13:**
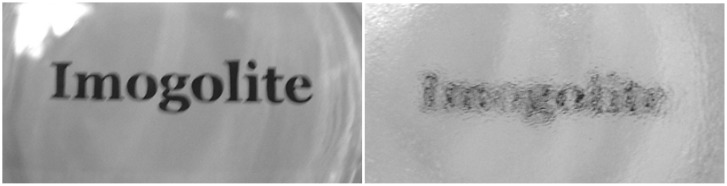
Transparency of *in situ* nanocomposite (left) and blend (right) films with imogolite:PVA = 1:20. The film thickness is circa 100 μm. Reprinted with permission from Ref. [56]. Copyright 2005 Royal Society of Chemistry.

As PVA can bind onto the surface of imogolite *via* hydrogen bonding, the interaction between the PVA and imogolite will have some effect on the optical properties of nanocomposites. To test this assumption, two kinds of imogolite/PVA films prepared by different methods were used for light transmission and haze value measurements [56]. One was prepared by simple blending of PVA with freeze dried imogolite powder [64] and the other was prepared by the previously mentioned *in situ* method with different weight ratios of imogolite/PVA. Obviously, the optical properties summarized by light transmittance (Figure 12) and haze value (Table 4) showed that the imogolite/PVA film prepared from the *in situ* method had a higher transparency than the blend one of the same imogolite/PVA weight ratio (1:20). It appears that stronger interactions resulting in intimate mixing of *in situ* growth imogolite with PVA is responsible for higher transparency compared to the blend one. *In situ* growth of imogolite that homogeneously dispersed within the matrix reduced the PVA crystallite size by diminishing the PVA crystallinity [56]. In contrast, blend sample of imogolite/PVA with existence of both PVA crystallite and imogolite aggregate show very strong anisotropic behavior [60], where both PVA crystallite and imogolite aggregate was large enough to scatter incoming light. Figure 13 shows a clear distinction between the transparency of *in situ* prepared and blend films with imogolite:PVA = 1:20. High optical clarity of this *in situ* imogolite/PVA nanocomposite may allow its potential application in paper coating [65], one of the common applications of pure PVA.

**Table 4:** Haze value of *in situ* synthesized imogolite/PVA nanocomposite, imogolite/PVA blend, and PVA films.

Sample (weight ratio)	Haze value/%
PVA	1.59 ± 0.19
Imogolite : PVA 1:20	1.00 ± 0.08
Imogolite : PVA 1:20	0.57 ± 0.02
Imogolite : PVA 1:20	2.55 ± 0.08
Imogolite : PVA 1:20^a^	29.94 ± 3.60

^a^ Blend film.

**Figure 14:**
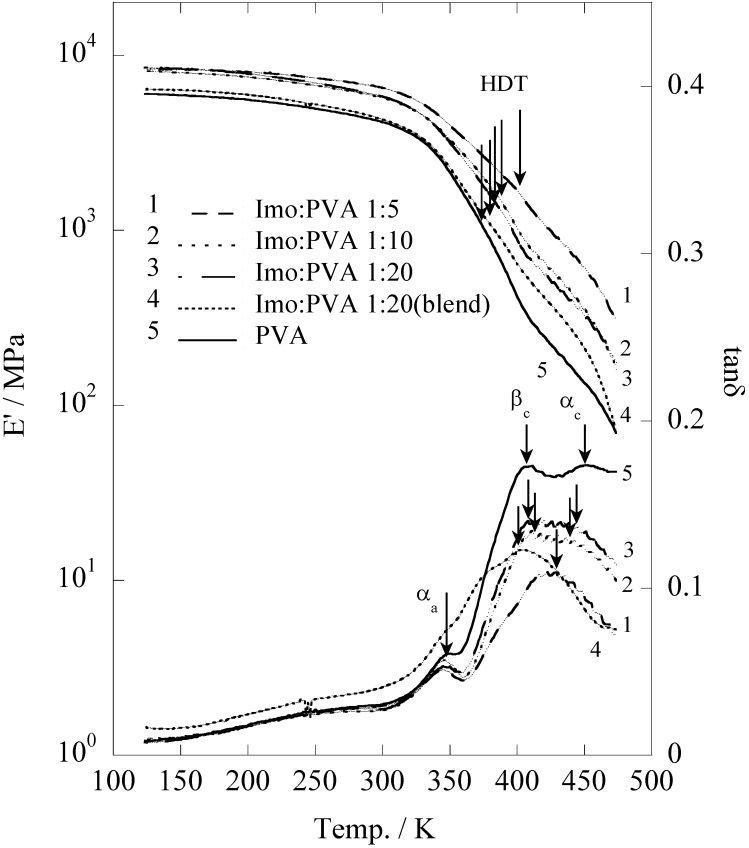
Temperature dependence of storage modulus and loss tangent plots of the *in situ* synthesized imogolite/PVA nanocomposite, imogolite/PVA blend and PVA films. Reprinted with permission from Ref. [56]. Copyright 2005 Royal Society of Chemistry.

The storage modulus (E'), which describes the elasticity of the pristine PVA, *in situ* prepared and blend film of PVA/imogolite, was evaluated by dynamic viscoelasticity (Figure 14) as a function of temperature ranges from 123 to 473 K [56]. Corresponding to the pristine PVA, the E' of the *in situ* and blend film of PVA slightly decreased with rising temperature and suddenly dropped around 325 K. It appears that the dynamic viscoelasticity curve of both *in situ* synthesized and blend film were identical to that of pristine PVA. Heat distortion temperature which defined as an index of heat resistance toward applied load was used to characterize the mechanical properties of the samples [66]. Generally, HDT values can be estimated from the storage modulus data at a point where the temperature at which the E' drops to 25% relative to its room temperature. Storage modulus (E') and HDT values of the sample were summarized in Table 5. The HDT of imogolite/PVA is strongly dependent to the imogolite content [67,68]. There is an ample increase in HDT, from 374 K for the pristine PVA to 402 K for *in situ* prepared imogolite/PVA (1:5). Interestingly, HDT of *in situ* prepared imogolite/PVA (1:20) was found to be slightly higher than that of the blend sample, probably due to a better interfacial interaction formed between PVA and *in situ* growth imogolite *via* hydrogen bonds. On the other hand, the improved mechanical properties of *in situ* prepared imogolite/PVA nanocomposites that accompany with increased imogolite content were attributed to the increased reinforcement effect. The bottom part of Figure 14 shows the changes of loss tangent (tan *δ*) as a function of temperature. Sample with smaller value of tan *δ* has more elastomeric behavior. The loss tangent curve of both *in situ* and blend imogolite/PVA is quite different from that of pristine PVA with a profound change was observed in the *β_c_* and *α_c_* absorption. Both *β_c_* and *α_c_* that assigned to the anharmonic twisting vibration and translational modes of chain segments along the chain axis, respectively, describe the chain motion within the crystalline phase of PVA [69]. We attributed the changes of *β_c_* and *α_c_* absorption to the alteration of twisting vibration and translation mode of PVA chains, respectively, when it forms hydrogen bonding with the imogolite surface [56].

**Table 5:** Room temperature modulus and heat distortion temperature of *in situ* synthesized imogolite/PVA nanocomposite, imogolite/PVA blend, and PVA films estimated from dynamic viscoelasticity measurement.

Sample	E'/GPa ^b^	HDT/K
PVA	4.2	374
Imogolite : PVA 1:20	5.8	383
Imogolite : PVA 1:20	5.8	388
Imogolite : PVA 1:20	6.6	402
Imogolite : PVA 1:20^a^	4.3	379

^a^ Blend film. ^b^ Evaluated at 298 K.

## 6. Functional Biocatalytic (Imogolite/Enzyme) Hybrid

Material-enzyme hybrids have attracted increasing attention for the development of next generation biosensor, active biological coating, biotransformation and therapeutics [70,71,72,73]. Immobilization of enzyme on a solid support will offer advantages for both industrial and analytical purposes, for instance, eliminating the risk of sample contamination, simplifying sample handling, as well as better separation of enzyme from solution that contains substrate and product [74]. As a nanoscale support, nanotubes of unique physicochemical properties represent an interesting material for stable immobilization of enzyme. Since then, enzyme has been successfully immobilized onto the carbon nanotube surface *via* covalent and non-covalent interactions [75,76,77]. However, the water-insolubility of carbon nanotube has severely limited their attractiveness for biotechnological applications, since almost all enzymatic reactions in animal cells occur in bodily fluid containing mostly water [78,79]. While there is a method that can convert carbon nanotube surface to be more hydrophilic by H_2_SO_4_:HNO_3_ treatment [80], issues concerning the stability, reusability and biocompatibility of this enzyme-carbon nanotube hybrid have yet to be investigated in detail. On the other hand, imogolite with its high surface area, chemical stability, water solubility, along with its better biocompatibility, could be of interest to substitute carbon nanotube as a robust support for enzyme immobilization. Moreover, the three-dimensional network of imogolite hydrogel formed in aqueous solution [81] can significantly increase the loading for enzyme immobilization and provide a scaffold for a much controlled enzymatic activities. High enzyme loading, solubility coupled with high stability and near native enzymatic activity of this material could thus satisfy the ultimate practical use of nanotube-enzyme hybrid.

Pepsin, a digestive enzyme secreted from the gastric mucosa, has a phosphoric acid group that can bind strongly to the imogolite surface, and as such provides a suitable model system for characterization of aggregation structure and enzyme reaction with imogolite [82,83]. Considerable studies have shown that immobilized pepsin (by methods including covalent binding, crosslinking) was able to hydrolyse various proteins such as serum albumin [84], albumin [85], hemoglobin [86], immunoglobulins [87] or casein [88]. There are few reports on the enzymatic reaction of immobilized pepsin at its optimum conditions (pH 3, 37 °C) and most of the studies were carried out in batch reactions with short-term reusability. In this review, we report a novel method of immobilization using the strong interaction between phosphoric acid groups of pepsin at the serine residue 68 with the Al-OH groups of imogolite [89]. The non-covalent interaction between the pepsin and imogolite is more favorable, as covalent interaction tends to disrupt the typical enzymatic activities by altering the chemical structure and reducing the conformational changes of pepsin that are critical for interacting with the substrate [90,91]. This imogolite/pepsin hybrid is expected to be stable enough to work repeatedly in prevalent conditions (pH 3, 37 °C) found in the stomach, and to be cheap enough to be exploited in proteomic research or industry at pilot plant scale

**Figure 15:**
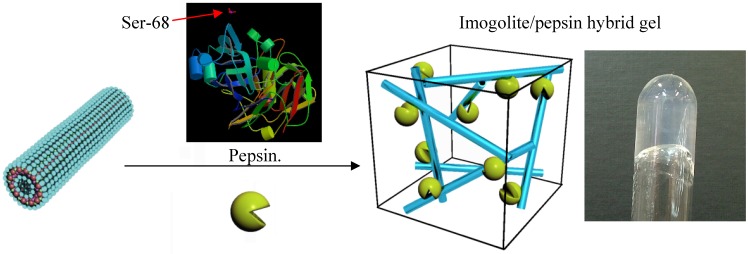
Schematic representation and photograph of imogolite/pepsin hybrid hydrogel.

Preparation and experimental detail of the imogolite/pepsin hybrid are described in literature [89]. Both 10 mL pepsin (1 mg/mL) and imogolite (0.5 mg/mL) acidic solution (pH 3.1) were mixed together, and the mixture was incubated at 37 °C (310 K) by shaking at 120 rpm for 4 h. The hybrid hydrogel imogolite/pepsin was collected by centrifugation of the mixture at 3000 rpm for 15 min and rinsed with acetic acid solution (pH 3.1) three times. Hybrid hydrogel of the imogolite/pepsin and the schematic representation of the three-dimensional network of imogolite/pepsin are shown in Figure 15. Under acidic condition, positively charged Al-OH group of imogolite surface generate electrostatic repulsion among nanotubes, which facilitate imogolite dispersion. Since almost every imogolite nanotube is surface exposed, we reasoned that high enzyme loading could be realized under acidic condition. Concurrently, pepsin becomes negatively charged at pH 3.1 since it has an isoelectric point of 1.0 [92]. Immobilization of enzyme can be achieved by forming a strong electrostatic interaction between positively charged imogolite and negatively charged pepsin. However, due to the high complexity of the protein structure, the immobilization behavior of pepsin onto imogolite surface is not completely understood. Other forces such as hydrogen bonding and hydrophobic interactions may also be hypothesized to be additional driving forces for synergy effect of pepsin immobilization [90].

**Figure 16:**
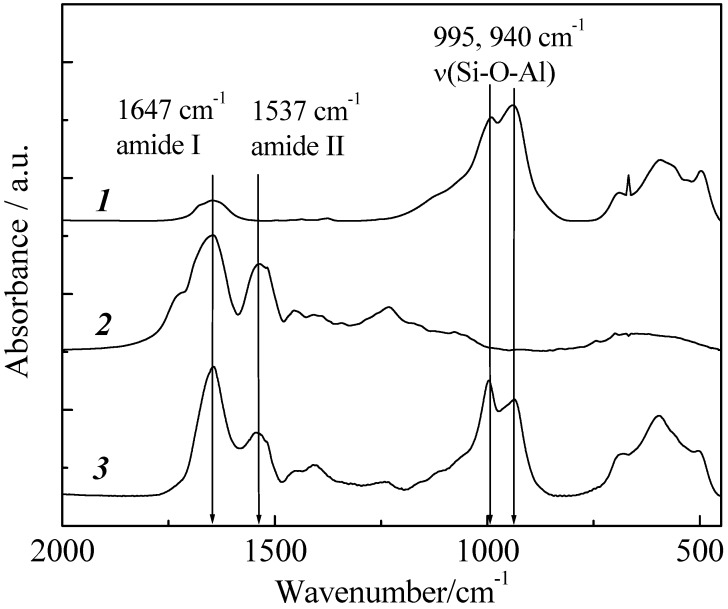
FT-IR spectra of (1) imogolite, (2) pepsin, and (3) imogolite/pepsin hybrid. Reprinted with permission from Ref. [89]. Copyright 2006 The Chemical Society of Japan.

Immobilization of pepsin on imogolite was spectroscopically verified by the FT-IR method. FT-IR is a very useful tool for determining the stability as well as functional groups of protein in complex. FT-IR spectra of imogolite, pepsin and imogolite/pepsin hybrid are shown in Figure 16. As mentioned above, Si-O-Al of imogolite usually absorbs at 995 and 940 cm^-1^ [49], while absorption peaks of amide I and II for pepsin can be observed at 1647 and 1537 cm^-1^, respectively [93]. For imogolite/pepsin hybrid, typical absorptions of both imogolite and pepsin were detected, confirming successful immobilization of pepsin on imogolite surface. Additionally, one can observe that the immobilization of pepsin with imogolite led to changes in absorption intensity ratio for both amides and Si-O-Al groups, respectively. Nevertheless, the position of absorption peaks for amide I and amide II in imogolite/pepsin spectrum are identical to that of pristine pepsin, indicating that the chemical structure of pepsin was not altered by the electrostatic interaction with imogolite.

**Figure 17:**
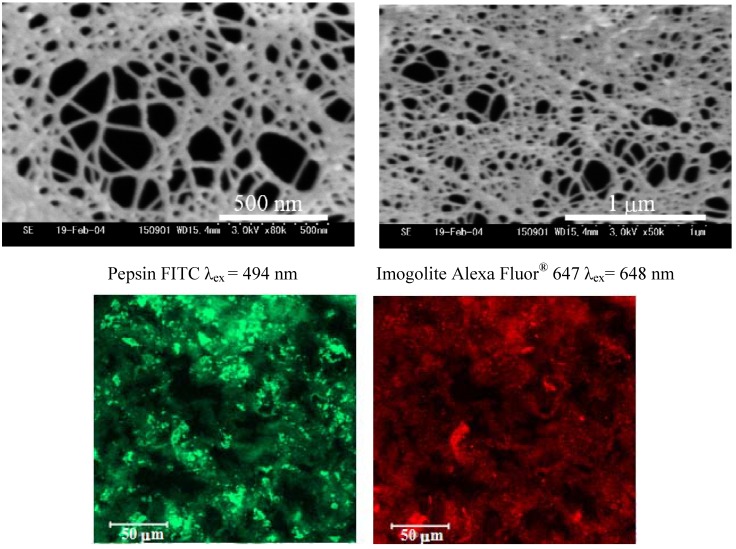
FESEM micrographs of imogolite/pepsin hybrid hydrogel (upper) and CLSM images of the sample (bottom). Reprinted with permission from Ref. [89]. Copyright 2006 The Chemical Society of Japan.

The amount of pepsin immobilized on the imogolite surface was calculated by TGA measurement. To obtain the solid product for TGA measurement, imogolite/pepsin hybrid hydrogel was subjected to freeze drying in toluene at 357 K (azeotropic temperature between H_2_O and toluene) followed by vacuum drying to remove the toluene residual. The maximum amount of immobilized pepsin was estimated to be 1.8 mg per 1 mg of imogolite, relatively higher than that of siliceous mesoporous material SBA-15/pepsin hybrid system [94]. Using this value to calculate the enzyme coverage on a single imogolite nanotube, we found that almost 71% of the imogolite surface was covered by pepsin molecules. The morphology of imogolite/pepsin hybrid hydrogel and the dispersion state of pepsin were visualized by field emission scanning microscopy (FESEM) and confocal laser scanning microscopy (CLSM), respectively. FESEM micrographs of imogolite/pepsin hybrid hydrogel (Figure 17, upper) clearly shows the presence of three-dimensional structures of imogolite/pepsin hybrid, with an average pore of hybrid hydrogel estimated to be circa 108 nm. For CLSM observation,: imogolite and pepsin were labeled with fluorescein 5-isothiocyanate, FITC, and adenosine 5'-triphosphate, Alexa Fluor^®^ 647 2'-(or-3')-*O*-(N-(2-aminoethyl)-urethane, hexa(triethylammonium) salt, respectively. From the fluorescent images, it was observed that imogolite/pepsin show similar morphology (Figure 17, bottom) with pepsin finely dispersed in the hybrid hydrogel.

**Figure 18:**
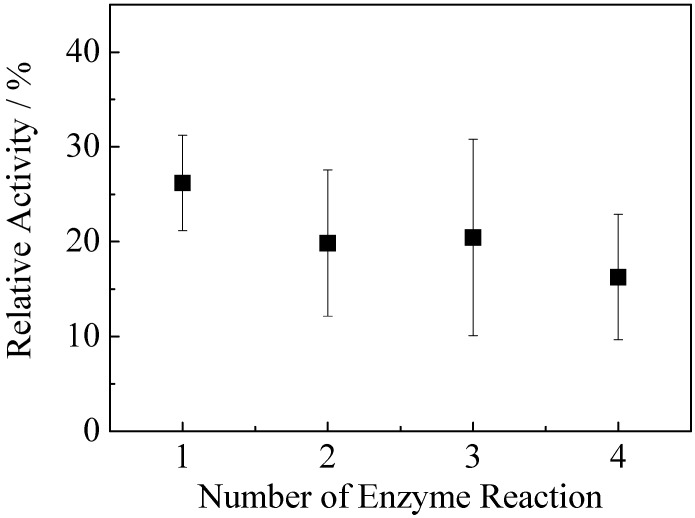
The enzyme activity change of immobilized pepsin during the repeated reaction at 310 K. Reprinted with permission from Ref. [89]. Copyright 2006 The Chemical Society of Japan.

The catalytic activities of the free pepsin and imogolite/pepsin hybrid were evaluated by testing peptic hydrolysis of a hemoglobin solution at pH 3.1. ΔA_280_/min of free pepsin measured by UV-vis spectroscopy was 0.183, and that of imogolite/pepsin hybrid was 0.048. Estimating the catalytic activity of pepsin at 100%, the activity of the imogolite/pepsin hybrid toward hemoglobin was circa 26%, a quarter lower than that of free pepsin. Hemoglobin is a bulky molecule of 68 kDa, 574 amino acid residues, and dimensions of 68 × 72 × 115 Å [95]. Judging from the FESEM images, the pore created by the imogolite/pepsin hydrogel may not be large enough to allow rapid diffusion of hemoglobin to the active site of pepsin for subsequent protein degradation activity. We hypothesized that only the pepsin molecules located at the external surface of three-dimensional network of hydrogel and those of leached pepsin molecules were actually interacting with hemoglobin. Similar report on reduced catalytic activity for Doulite/pepsin [96] and SBA-15/pepsin [94] indicate that immobilized pepsin still faces disadvantages such as deprived catalytic activity and restricted substrate, intermediate and product diffusion. Finally, the reusability of this imogolite/pepsin hybrid hydrogel was tested up to four catalytic cycles (Figure 18). The hybrid hydrogel retained 75% of activity up to three cycles, then the activity reduced to 60% after four cycles. This result implies that some weakly bound pepsin leached at the first cycles of catalytic reaction which led to a noticeable decrease of catalytic activity. The remaining strongly bound pepsin retained its catalytic activities after two and three cycles. After three cycles, almost 40% of enzyme amount was leached out, resulting in further decreased catalytic activity after four cycles.

To the best of our knowledge, this is the first study of immobilized pepsin catalytic activity on inorganic nanotube *via* non-covalent electrostatic interactions. This novel system exhibits reduced catalytic activity due to the incompatible of hemoglobin dimensions with the hybrid hydrogel pore. It was also speculated that the electrostatic interaction of pepsin on imogolite is not strong enough to endure repeated catalytic reaction. However, it is worth to reinvestigate its catalytic activity using smaller dimension substrate such as Z-Glu-Tyr dipeptide [94]. The Z-Glu-Tyr will have less steric hindrance and thus could interact with pepsin hidden between the nanotube intervals.

## 7. Multilayered Composite Film Composed of Water-Soluble Conjugated Polymer and Imogolite

In recent years, transparent conductive films composed of carbon nanotube and organic polymer on non-conductive substrate have attracted much interest due to their essential application in electronic devices including touch panels, sensor, photovoltaic cell, electrochromic device, flat or flexible electronic display [97,98]. Transparent conductive films composed of carbon nanotube and organic polymer matrix have many advantages over conventional metal coated glass such as Sn-doped In_2_O_3_ (ITO) substrate [99]. With energy intensive fabrication processes during metal deposition on substrate by mean of electron beam evaporation or sputter deposition aggravated by scarcity of rare metal elements, the carbon nanotube/organic polymer matrix composite film is high in demand in the future for the sake of light weight, low cost and flexibility.

Controlling the architecture of nanotube based thin films at nano-scale level for tailored film properties and functionality by simple coating and printing technology has been widely explored. Kovtyukhova *et al.* have reported anisotropic conductivity behavior of oxidized single-wall carbon nanotube/polyaniline (SWNT/PANI) film prepared by layer-by-layer (LBL) assembly technique, where two oppositely charged components are grown layer-by-layer onto the substrate [100]. This sub-nanometer thick PANI layer interweaved with the monolayer of SWNT exhibiting the maximum electrical conductivity of 5 × 10^2^ S/m. However, the film suffered from oxidation, which destroyed the conjugation of the aromatic ring of SWNT, thus drawing down the overall electrical conductivity. In this context, Adachi *et al.* prepared a stable water-soluble conjugated polymer poly(p-phenylene ethylene) (PPE)/SWNT LBL composite film using non-covalent π-π interaction [101]. The conductivity of LBL composite films is significantly enhanced by several orders of magnitude (4.9 × 10^-3^ S/m) compared to that of pristine PPE film (8.3 × 10^-9^ S/m) with anisotropic electrical conductivity depending strongly on SWNT-aligned direction. Although carbon nanotube is a promising material for design of functional thin films, mainly because of its excellent electrochemical properties, limitations exist in processability of carbon nanotubes in solution; especially in water or other common solvents, and thus greatly hinders their application in LBL film. Attempts to oxidize carbon nanotubes have been made to provide charges on the surface for the purpose of water dispersion. Nevertheless, concern over the destruction of the carbon nanotube conjugation system, which would impair the electrical conductivity, should be taken into serious considerations. Furthermore, due to the high conjugation system of carbon nanotubes, it is very difficult to obtain a dense film without sacrificing the optical transparency of the film. With regard to these problems, we have focused on imogolite as a potential alternative to carbon nanotube for a stable and highly transparent multilayer nanotube/polymer composite thin film.

The grand challenge of nanotube based thin film is distributing imogolite homogeneously at monomolecular level in polymer connecting matrix, as imogolite tends to aggregate into bundles *via* hydrogen bond and van der Waal forces. A well known and common way of assembling the dispersed nanotube would be the LBL method, which consists of sequential deposition of nanometer thick layers of nanotube and polymer to achieve conformal ultrathin film with tunable surface properties [102]. Deposition is done by repeated and sequential immersion of a substrate into aqueous solutions of oppositely charged materials with rinsing to remove weakly adsorbed materials followed by drying after each immersion. Another interesting approach to achieve similar results is spin-assembly. This technique was developed based on the LBL assembly method where deposition on the substrate done by the immersion step is taken over by spin coating process [103,104]. Using the spin-assembly method, the weakly adsorbed components and additional solvent can be removed by centrifugal force during spinning. thus eliminating the need of intermediate rinsing and drying steps [105,106]. Additional advantages of spin-assembly over LBL assembly include time efficiency, reproducibility, flexibility with high tunability in terms of deposition amount [107].

**Figure 19:**
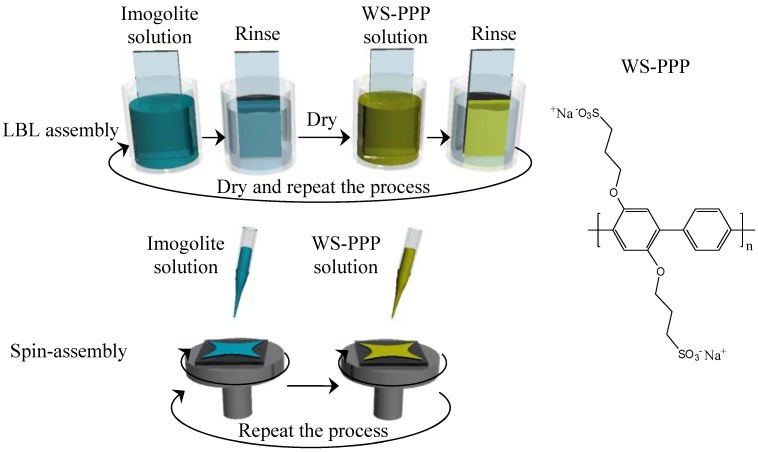
Schematic representation of LBL and spin-assembly as well as chemical structure of WS-PPP.

The ability to generate sufficient electric field and emit electrons at the tip of the carbon nanotube is believed to be due to its high aspect ratio and geometry [108]. It is intriguing to think that imogolite, which shares similar geometry and tubular structure with carbon nanotube, may also be a good electron emitter as well. Oh *et al.* studied the current-voltage characteristics of imogolite and observed current flow upon exposure to water. The current observed might be due to the effective motion of protons (H^+^), which involves the formation and destruction of bonds through a long chain of water molecules adsorbed on the imogolite surface [108]. We expect that imogolite, when exposed to aqueous solution, may not simply act as a positively charged counterpart for LBL or spin-assembly, but also facilitate electric flow for fully conductive coating. In this review, we report the fabrication of novel multilayer imogolite/conjugated polymer composite films by applying LBL and spin-assembly methods. Here, negatively charged water-soluble conjugated polymer poly[disodium 2,5-bis(3-sulfonatepropoxy)-1,4-phenylene-*alt*-1,4 -phenylene] (WS-PPP) as shown in Figure 19 and positively charged imogolite were alternatively absorbed on oxidized 3-mercaptopropyltrimethylsilane (MTS) processed substrate. This imogolite-based multilayer film is not only conductive, but also highly transparent - a winning combination of parameters for high performance optoelectronics, energy conversion and storage. These resulting multilayer imogolite/WS-PPP composite films prepared by two different assembly methods were characterized and compared.

The preparation of imogolite/WS-PPP composite film by LBL and spin-assembly was described elsewhere [107]. Silicon substrates and quartz plate for the preparation of composite films were treated with MTS *via* a CVA process to coat the silicon surface with a monolayer of mercapto functionalized organosilane [109]. By subsequent oxidation, the mercapto groups can be converted to negatively charged sulfonic acid groups, which is essential for the initial deposition of imogolite [110]. Fabrication of homogeneous imogolite/WS-PPP composite film must start from the preparation of well dispersed imogolite solution. Solutions of sodium acetate and acetic acid were used to prepare an acetate buffer pH 4 as the imogolite solvent. Under acidic conditions, isolated units of positively charged imogolite nanotube can be retained that give homogeneous dispersion of imogolite in the solution. In the case of LBL assembly, the negatively charged substrate was dipped into imogolite solution (1 mM, 20 min) and WS-PPP solution (1 mM, 10 min), alternatively. Deposition of each component was followed by rinsing with water for 2 min and a drying step. The deposition of imogolite and WS-PPP onto the substrate by LBL assembly is illustrated in Figure 19. For spin-assembly, deposition was done by dropping circa 0.5 mL of imogolite solution onto sulfonated substrate while spinning at 6000 rpm for 20 s. A similar step was applied to the WS-PPP solution for second layer deposition. The process, as shown in Figure 19, was repeated to make multilayer films.

**Figure 20:**
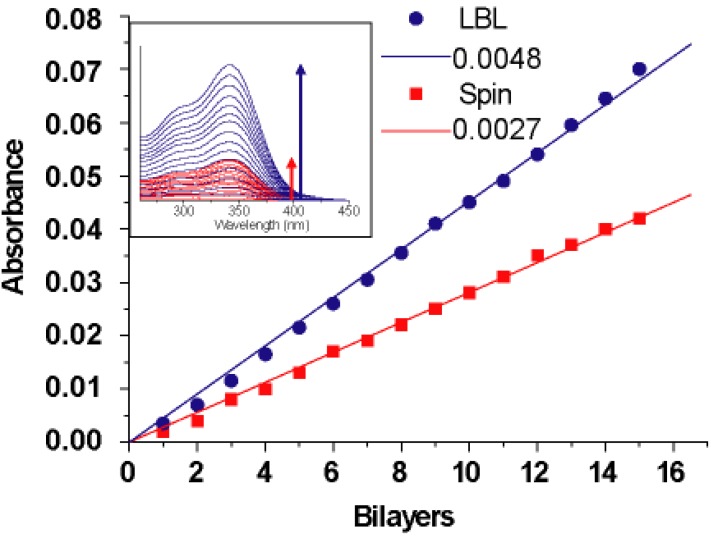
Increase in absorbance at 345 nm of 15 bilayer films prepared by LBL and spin-assembly. The inset shows the UV-vis absorption spectra of WS-PPP deposited on the films. The slope implies the absorbance unit per bilayer (abs unit/bilayer). Reprinted with permission from Ref. [107]. Copyright 2008 The Chemical Society of Japan.

The formation of multilayer films containing imogolite and WS-PPP prepared by LBL and spin-assembly were monitored by UV-vis spectroscopy. Figure 20 shows the UV-vis spectral change of both LBL and spin-assembly films prepared from 1 to 15 layers on a MTS treated quartz substrate. The absorption peaks for both LBL and spin-assembly films increased with the number of layers, and the absorption intensity at λ_max_ = 345 nm (Figure 20, inset) attributed to the WS-PPP backbones increased linearly with the number of layers. It should be noted for LBL assembly that deposition of material occured on both side of the substrate during the immersion step, compared to spin-assembly where only one side of the substrate was used for spin-coating. Thus, to better compare the deposition behavior of LBL and spin-assembly, absorbance values of the LBL assembly were divided by half for normalization [107]. The average increase in the absorption intensity was approximately 0.0048 and 0.0027 absorbance units per bilayer for LBL and spin-assembly, respectively. Direct measurement of the average hybrid film thickness using the AFM method were obtained by scratching the hybrid films with a razor blade to reveal the height difference between the film region and scratched substrate side. Thickness measurements were carried out at several points and the average film thickness was 2.4 nm per bilayer for LBL and 1.7 nm for spin-assembly. As demonstrated by both UV-vis absorbance and direct AFM measurement, the absorbed amount of WS-PPP and imogolite using LBL method is higher than that of the spin-assembly. Such a difference in absorption amount is implicated by the mechanism each LBL and spin-assembly exerts. In LBL assembly, imogolite and WS-PPP of opposite charges need a longer time to settle on the substrate surface during the dipping and pulling processes, which results in a near equilibrium state of absorption. For spin-assembly, however, most of the imogolite and WS-PPP are spun away by centrifugal and shear air force, which complicates the equilibrium state for a higher amount of absorption. Despite a lower component absorption capability, spin-assembly could still provide smoother with thicker film (increase the spinning cycles) without much difficulty.

The controlled nanotube alignment on the substrate is critical to fabricate high performance device with versatile functionality. Particularly, planar aligned nanotubes on suitable substrate enables integration of nanotube on circuit for high efficiency nanotube-based electronics [111]. There are several methods so far being developed to control and align the carbon nanotube on substrate, which include wet spinning [112], cooperative reorientation of a liquid crystal carbon nanotube suspension in electric field [113], magnetic orientation in conducting polymer matrix [114,115,116], CVD growth on trenched substrate [111], Langmuir-Blodgett [117] and LBL assembly [118]. However, most of these approaches are often tedious, time consuming, possess the possibility of nanotube degradation and suffer from a low degree of alignment as well as low density of carbon nanotube. Despite these issues, orientation of imogolite that exhibit tubular morphology comparable to that of carbon nanotube using the above mentioned method has rarely been reported. Here, a top-down based approach for potential large scale-controlled of dense imogolite nanotube alignment on silicon substrate using simple spin-assembly method is presented.

**Figure 21:**
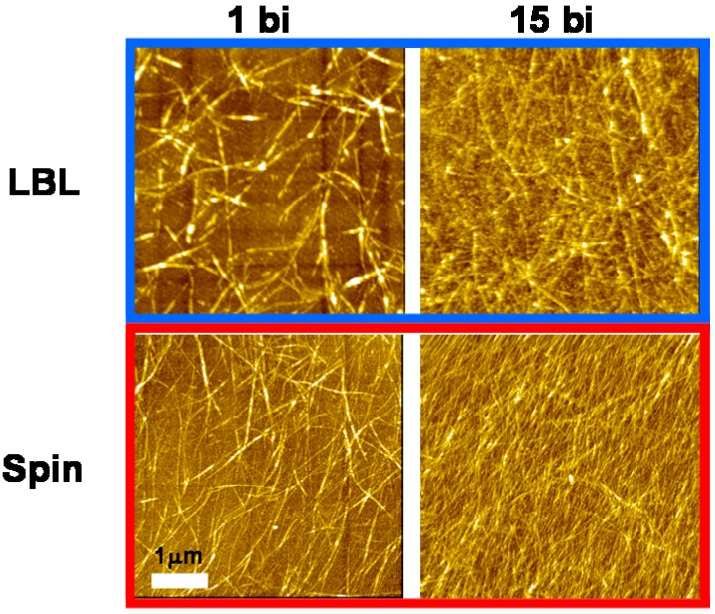
AFM images of 1 and 15 bilayer films prepared by the LBL (top) and spin-assembly (bottom). Reprinted with permission from Ref. [107]. Copyright 2008 The Chemical Society of Japan.

Planar alignment of imogolite nanofiber interwoven with the WS-PPP monolayer using the spin-assembly method was observed by AFM. The AFM images of the LBL and spin-assembled imogolite on silicon substrate are shown in Figure 21. The AFM images of imogolite nanofiber in 1 and 15 bilayers films, strongly demonstrating that the spin-assembled imogolite nanofibers are better aligned than the LBL-assembled. Interestingly, well aligned imogolite nanofiber was observed even at a thicker spin-assembled film (15 bilayers). The spin-assembled imogolite/WS-PPP multilayered composite film was careful examined by the AFM method to give an insight into the effect of spin-assembly on the alignment of this positively charged imogolite.

**Figure 22:**
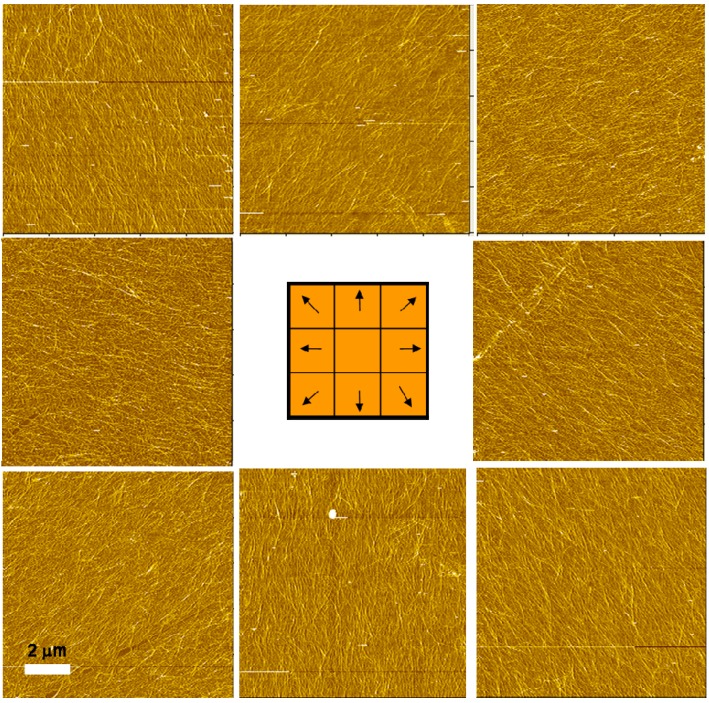
AFM images of imogolite nanofiber films at eight cardinal points. Reprinted with permission from Ref. [107]. Copyright 2008 The Chemical Society of Japan.

The AFM images presented in Figure 22 show the surface morphology of spin-assembled imogolite nanofiber at eight cardinal points. Such orientation is believed to be ascribed to shear force and centrifugal force exerted during the spinning process that pull the nanotube outward in a radial direction before they settle by forming electrostatic interaction with the former polymer layer. Accordingly, due to the minor shear force and centrifugal force in the center area of substrate, evolution of radial aligned imogolite nanofibers did not occur [107].

**Figure 23:**
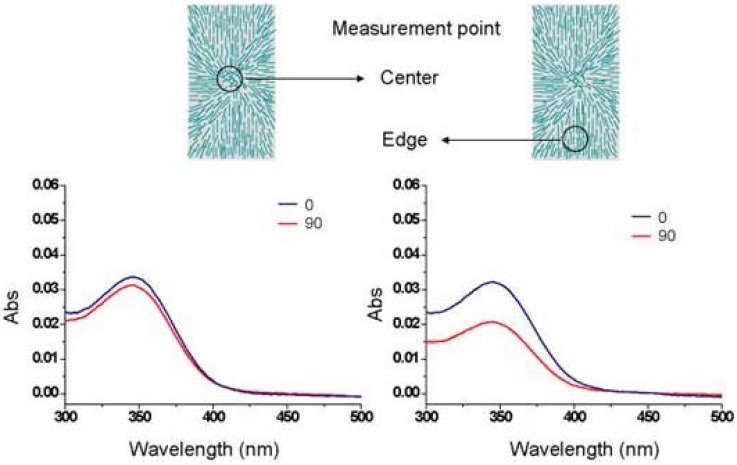
Schematic representation of imogolite nanofibers on the spin-assembled substrate and polarized UV-vis absorption spectra of 15 bilayers spin-assembled film measured at center (left) and edge (right) area of the substrate. Reprinted with permission from Ref. [107]. Copyright 2008 The Chemical Society of Japan.

Polarized absorption spectra of spin-assembled film on glass plates were recorded on a UV-vis-NIR scanning spectrometer (Shimadzu UV-3150) using a polarizer (Assy II; 260–2500 nm) and a sample-immobilization set of glass plates (Shimadzu P/N 206–81042) [116]. Since the imogolite alignment differed in cardinal direction, two measurement points (shown in Figure 23) were targeted by a narrow beam spot of 4 mm diameter. UV-vis light (300–500 nm) was polarized in the direction parallel (||; 0°) and perpendicular (⊥; 90°) to the rotation axis of substrate. Due to randomly oriented imogolite on the center area of the substrate, the absorbance at 345 nm was almost identical in both polarization directions (parallel and perpendicular). On the other hand, at the edge area, the absorbance (A) of the spin-assembled imogolite on the glass plate parallel to the polarized light (||; 0°) was higher than that of perpendicular direction (⊥; 90°) to the extent of A_||_/A_⊥_ = 1.53. In the case of LBL film, the absorbance of both direction A_||_ and A_⊥_ were almost same in any position on the substrate. The polarized absorption spectra result is in agreement with the previous described AFM image that shows the anisotropy behavior of imogolite in spin-assembled film. The creation of densely packed WS-PPP layer with controlled imogolite alignment on substrate makes the spin-assembly technique attractive for nanotube based electronic that can be exploited for mass production.

## 8. Conclusions

In this review, imogolite based nanocomposites material prepared using various methods has been discussed. Organophosphonic acid group appears to be an effective coupling agent to modify the external surfaces of imogolite to allow better dispersion in the matrix polymer. Transparent PMMA nanocomposites containing P-HEMA modified imogolite were prepared by a polymer grafting method. Mechanisms such as homogenous dispersion, barrier effects of imogolite and stronger interfacial interaction with PMMA, are the causes of the improvement in thermal/mechanical stability and optical properties of PMMA/imogolite nanocomposites. The *in situ* synthesis method has shown to be powerful capability for the creation of homogeneous and reproducible imogolite nanotube within the PVA polymer matrix. As was pointed out, precise control over the uniform dispersal of imogolite within the polymer matrix is crucial for achieving efficient load transfer and higher optical transparency of nanocomposites. Nanocomposite containing this *in situ* growth imogolite nanotube shows higher optical transparency and excellent mechanical properties compared to those blended. Novel soluble hybrids by attaching enzyme to imogolite using non-covalent interaction was developed. The high support surface area facilitates the preparation of imogolite/pepsin hybrid with high enzyme loading. The imogolite/pepsin hybrid described here represents a new class of highly uniform protein hybrid system that is active, stable, and reusable as well as being water-soluble and easily recoverable. Finally, we have demonstrated the technique of preparing transparent conductive films composed of imogolite and water soluble conjugated polymers WS-PPP with anisotropic behavior within the film. This is a convenient and versatile technique for the alignment of imogolite-polymer composites based on spin-assembly. The transparent and anisotropic spin-assembled film appears to have many potential applications at large scale. It is expected that imogolite technology, which underwent rapid development during the past decades, will be able to compete with their carbon nanotube counterpart in the future. Of course, there are still a lot of on-going problems in imogolite technology that need to be solved and research on more sophisticated imogolite systems is encouraged.
